# Adherence to antihypertensive medications in rural Lao PDR: a prospective observational study

**DOI:** 10.1186/s41182-021-00374-4

**Published:** 2021-10-29

**Authors:** Emiri Takahashi, Phoutnalong Vilay, Ketmany Chanthakoummane, Tiengkham Pongvongsa, Sengchanh Kounnavong, Shigeyuki Kano, Jun Kobayashi, Daisuke Nonaka

**Affiliations:** 1 Faculty of Pharmacy, Takasaki University of Health and Welfare, Takasaki, Gunma Japan; 2 Center of Malariology, Parasitology and Entomology, Ministry of Health, Vientiane, Lao PDR; 3 Lao Tropical and Public Health Institute, Ministry of Health, Vientiane, Lao PDR; 4 Savannakhet Provincial Health Department, Savannakhet, Lao PDR; 5 Faculty of Tropical Medicine, Mahidol University, Bangkok, Thailand; 6 Department of Tropical Medicine and Malaria, Research Institute, National Center for Global Health and Medicine, Tokyo, Japan; 7 Department of Global Health, Graduate School of Health Sciences, Faculty of Medicine, University of the Ryukyus, Okinawa, Japan

**Keywords:** Medication adherence, Hypertension, Laos

## Abstract

**Background:**

Although hypertension is becoming more prevalent among the adult population of the Lao People’s Democratic Republic (PDR), with a prevalence of approximately 20% in 2013, treatment adherence of patients with hypertension, especially those in rural areas, remains poorly understood. The objective of the present study was to examine the rate of medication adherence to antihypertensive medicines among outpatients with hypertension in rural districts of the Savannakhet.

**Methods:**

A prospective observational study was conducted in Xepon, Phin, and Nong districts. The study population was outpatients aged 18 years or older who were prescribed antihypertensive medicines at three district hospitals between February and August 2017. Data were collected on the first day of treatment (day 0) and the day of follow-up (around day 7) through interviews with the patients and outpatient registration books. The medication adherence rate was determined using the four-item Morisky Medication Adherence Scale. The level of adherence was evaluated by the sum of the scale, with scores ranging from 0 to 4 points. The adherent group, namely those with a score of 0, and the non-adherent group, namely those with scores of 1 to 4 points, were compared. Fisher’s exact test was used to identify factors associated with medication adherence.

**Results:**

Of the 68 patients examined, 38.2% newly began treatment. Half of the patients (*n* = 34, 50.0%, 95% CI 0.38 to 0.62) adhered to medication instructions. The adherent group was more likely to have received written instructions when prescribed medication, compared to the non-adherent group (79.4% vs 55.9%, *p* = 0.068). Those who perceived that hypertension needs lifelong treatment were significantly more likely to adhere to the medication regimen (*p* = 0.028).

**Conclusions:**

Medication adherence was present among 50% of outpatients with hypertension who visited a district hospital. Therefore, providing written instructions to patients would be effective for improving medication adherence.

## Background

Non-communicable diseases (NCDs) kill 41 million people yearly, accounting for 71% of global deaths, of which approximately 15 million are “premature”, which indicates death among individuals aged 30–69 years. Of premature deaths, 85% occur in low- and middle-income countries [1]. Among non-communicable diseases, high blood pressure is associated with one of the highest risks of premature death [2–5].

The International Society of Hypertension, 2020 Global Hypertension Practice Guidelines [6] recommend beginning pharmacologic treatment for patients with blood pressure > 140/90 mmHg, for whom lifestyle treatments have not been effective. Patients with blood pressure ≥ 160/100 mmHg are recommended to start pharmacologic treatment immediately alongside lifestyle interventions. Most patients who are prescribed pharmacologic treatment require more than one medication to control their blood pressure. Medication is chosen based on age, ethnicity/race, and other clinical characteristics (e.g., comorbidities). The treatment goal for systolic blood pressure (SBP) is ideally < 140 mm Hg and < 90 mm Hg for diastolic blood pressure (DBP).

Poor adherence to antihypertensive therapies has been linked to various problems, including poor blood pressure control, rehospitalization, and increased healthcare resource utilization [7–10]. A systematic review showed that the level of adherence to cardiovascular medication according to self-report was 54.6% (95% confidence interval 47.7% to 61.5%) in resource-limited settings [11].

The Lao People’s Democratic Republic (Lao PDR) is in South-Eastern Asia. Lao PDR has been affected by a double burden of disease, that is, the existence of both communicable and non-communicable diseases—as is the case in other low- and middle-income countries. In Lao PDR, non-communicable diseases (NCDs), including hypertension and diabetes, have recently become prevalent [1, 12]. The “national multisectoral action plan for the prevention and control of non-communicable diseases 2014–2020” stated, “Non-communicable diseases are effectively and equitably prevented and controlled for people in Lao PDR, and the burden of non-communicable diseases on households and society is minimized” [14]. In Lao PDR, the provision of integrated management of NCDs through primary care was one of the priority actions. To prevent and control NCD, several activities (e.g., screening, counselling, treatment, follow-up, referral, and health education) were expected in health services at the primary healthcare level (i.e., health center and district hospital). Compared to the 2013 baseline prevalence of raised blood pressure, the government adopted a national target of a 15% reduction by 2020 (prevalence: 15.6%) and a 25% reduction by 2025 (prevalence: 13.7%) [13].

In a national cross-sectional study conducted in 2013, 20% of adults aged 18–64 years exhibited hypertension. Among such persons with hypertension, awareness of the condition was low, as was the proportion receiving treatment and proportion whose blood pressure was successfully controlled [14]. Suboptimal adherence is known to have a broad impact on health consequences (i.e., cardiovascular disease) through inadequately treated or untreated hypertension [10, 15].

However, few studies have explored adherence to antihypertensive medication in Lao PDR. Accordingly, this study examined adherence among patients with hypertension and identified its related factors in rural Lao PDR.

## Methods

### Study site and population

This study was conducted in the Xepon, Phin, and Nong districts of Savannakhet province, Lao PDR. These districts were selected because malaria remains endemic, and most healthcare resources are directed toward treating acute diseases; therefore, it is thought that NCDs are not adequately addressed. Furthermore, we thought it would be possible to compare the results of this study on adherence to antihypertensives with those of the study on adherence to anti-malaria medication [16]. Therefore, data were collected in the three district hospitals.

In rural areas of Lao PDR, health centers provide basic services for the treatment and prevention of common illnesses [17] and health promotion [18]. Meanwhile, district hospitals play a major role in providing treatment for NCDs at the primary level. District hospitals may prescribe ACE inhibitors, one of the essential antihypertensive drugs available in Lao PDR. The district hospitals were selected because the region has a double burden of communicable and non-communicable diseases; thus, district hospitals need to address this burden at the primary level.

Patients who met the following criteria were included: (1) patients who consulted the outpatient department (OPD) or emergency room (ER) between February and August 2017, (2) those who were diagnosed with hypertension and were prescribed oral antihypertensive medications by a physician, and (3) those who were 18 years of age or over.

### Data collection

Data were collected between February and August 2017. On the day of consultation (day 0), immediately after the patients completed consultation and examination, those who met the inclusion criteria were invited to participate in the study by a study physician. These physicians worked at the study hospital and agreed to cooperate with the study.

The study physicians observed how the dispensing staff gave medication instructions to the patients, including the characteristics of the dispensing staff and the language the patient spoke. The study physicians also interviewed patients using a questionnaire. The questionnaire collected contact details, information on socio-demographics, lifestyle, clinical history (i.e., history of medications prescribed, frequency of hospital visits, reasons for hospital visits, and physical condition), total expenditures for receiving treatment (including transportation to the health facility), and adherence to the antihypertensive medicines. In accordance with the clinical guidelines [19], people were classified into four groups based on their blood pressure level, normal blood pressure (< 130 SBP and DBP < 85 mmHg), high-normal blood pressure (130–139 SBP and/or DBP 85–89 mmHg), grade 1 (140–159 SBP and/or DBP 90–99 mmHg) and grade 2 hypertension (≥ 160 SBP and/or DBP ≥ 100 mmHg).

Adherence was measured using the four-item Morisky Medication Adherence Scale (MMAS-4) [20] (Table 1). The MMAS-4 comprises four items with dichotomous response options of “yes” or “no”. Because of its reverse wording of questions to address positive response bias, the instrument can obtain disclosures of non-adherence; furthermore, it captures the barriers to adherence. Since the instrument has been validated with a broad range of diseases and in patients with low literacy, it is commonly used for research [21–28]. For this study, a Laotian doctor, fluent in both English and Laotian, translated the questionnaire from English to Laotian. Following this, another Laotian doctor verified the accuracy of the translation. Finally, the face validity of the questionnaire was confirmed by surveyors (medical doctors).

**Table 1:** Morisky Medication Adherence Scale (MMAS-4)

Do you ever forget to take your medicine?	0. No, 1. Yes
Are you careless at times about taking your medicine?	0. No, 1. Yes
When you feel better, do you sometimes stop taking your medicine?	0. No, 1. Yes
Sometimes if you feel worse when you take the medicine, do you stop taking it?	0. No, 1. Yes

Some demographic and clinical information were collected from outpatient register records and consultation records, including age, sex, disease, prescribed medicines, and laboratory data.

On day 7, the study physicians collected the patient’s medication adherence data for the preceding 7 days, medication instructions the patients received on day 0, the patient’s comprehension of the dose, and the patient’s physical condition on day 7. These data were collected by phone or by face-to-face interview. If the study physicians were unable to collect data on day 7, data were collected on day 8.

### Data analysis

The level of adherence was determined by the summed score of the MMAS-4: a score of 0 indicated high adherence, a score of 1 or 2 denoted moderate adherence, and a score of 3 or 4 indicated low adherence. The adherent (high adherent group) was compared with the non-adherent (moderate and low adherence groups) in terms of socio-demographic characteristics, clinical characteristics, and content of medication instructions received and their perception. The Mann–Whitney *U* test and the Student’s *t*-test were used to compare counts or continuous variables, whereas the Fisher’s exact test was used for categorical variables. A *p*-value of < 0.05 was considered statistically significant. All analyses were performed with SPSS version 26.

## Results

During the study period, 119 patients met the inclusion criteria, of whom 75 were accessible and interviewed on day 0. Around day 7, 68 out of 75 patients were accessible and interviewed face-to-face at the study hospital (*n* = 34) or over the phone (*n* = 34). Of the 68 patients examined, 26 (38.2%) newly began antihypertensive treatment. Regarding the level of adherence, 34 showed high adherence, 22 medium, and 12 low.

Table 2 shows the socio-economic characteristics of the patients. Thirty-four patients (50.0%) were male. Furthermore, most of the patients could speak Lao and 50 (73.5%) completed at least primary school. The median age of the patients was 55 years old (interquartile range [IQR], 46.5 to 69.5 years).

**Table 2:** Socio-economic characteristics of the patients

	Adherent group (*n* = 34), *n* (%)	Non-adherent group (*n* = 34), *n* (%)	*p* value
Study site			
Nong	16 (61.5)	10 (38.5)	0.083^a^
Phin	13 (54.2)	11 (45.8)	
Xepon	5 (27.8)	13 (72.2)	
Sex (female)	19 (55.9)	15 (44.1)	0.47^a^
Age (years), mean (SD)	58.0 (11.9)	58.6 (18.8)	0.89^b^
Language			
Lao or Phouthay	32 (51.6)	30 (48.4)	0.67^a^
Others	2 (33.3)	4 (66.7)	
Ethnic group			
Lao (Lao-Tai)	17 (48.6)	18 (51.4)	
Mangkong (Mon–Khmer)	5 (38.5)	8 (61.5)	
Tri (Mon–Khmer)	1 (100)	0 (0)	
Phouthay (Lao-Tai)	5 (55.6)	4 (44.4)	
Data unavailable	6 (60.0)	4 (40.0)	
Educational attainment (highest education level)			
Less than primary school	9 (52.9)	8 (47.1)	0.86^a^
Primary school	12 (52.2)	11 (47.8)	
Secondary school or more	12 (44.4)	15 (55.6)	
Data unavailable	1 (100)	0 (0)	
Employment status			
Employed	7 (36.8)	12 (63.2)	0.28^a^
Not employed	26 (54.2)	22 (45.8)	
Data unavailable	1 (100)	0 (0)	
Health insurance			
Staff	11 (42.3)	15 (57.7)	
Company	2 (50.0)	2 (50.0)	
Low income	9 (69.2)	4 (30.8)	
Community	3 (60.0)	2 (40.0)	
None	9 (47.4)	10 (52.6)	
Data unavailable	0 (0)	1 (100)	
Means of interview on day 7			
Telephone	17 (50.0)	17 (50.0)	1^a^
Face-to-face	17 (50.0)	17 (50.0)	

^a^Fisher’s exact test

^b^Student’s *t*-test

Nineteen patients (27.9%) were employed and 49 (72.1%) had medical insurance. There were no significant differences in sex, age, language, education attainment, or rate of holding medical insurance between the adherent and the non-adherent groups.

Approximately one-third (33.8%) of patients were admitted after consultation. The major reasons for the consultation were headache (60.3%), dizziness (26.5%), nausea (25.0%), and tiredness (19.1%). Fifty-two patients (76.5%) were classified with grade 2 hypertension. Regarding NCD comorbidities, six patients had diabetes and one had heart failure (Table 3).

**Table 3:** Clinical characteristic of the patients

	Adherent group (*n* = 34)	Non-adherent group (*n* = 34)	*p* value
Direction after consultation			
Return home	22 (50.0)	22 (50.0)	1.0^a^
Admission	11 (47.8)	12 (52.2)	
Referral	1 (100)	0 (0)	
Body mass index (BMI), mean (SD)	24.1 (3.9)	24.0 (3.9)	0.90^b^
Normal (18.5–24.9)	23 (53.5)	20 (46.5)	
Underweight (below 18.5)	2 (66.7)	1 (33.3)	
Overweight (25.0–29.9)	8 (42.1)	11 (57.9)	
Obese (30.0 and above)	1 (50.0)	1 (50.0)	
Blood pressure			
Systolic blood pressure (SBP)	158.8 ± 21.7	164.3 ± 21.9	0.30^b^
Diastolic blood pressure (DBP)	98.3 ± 11.2	99.3 ± 11.5	0.70^b^
Classification of hypertension [19]			
Normal blood pressure	1 (100)	0 (0)	0.10 ^a)^
High-normal blood pressure	0 (0)	1 (100)	
Grade 1 hypertension	10 (71.4)	4 (28.6)	
Grade 2 hypertension	23 (44.2)	29 (55.8)	
Newly began antihypertensive medication	13 (50.0)	13 (50.0)	1.0^a^
Aware of hypertension			
Aware	21 (50.0)	21 (50.0)	
Ever treated (medication)	21 (50.0)	21 (50.0)	
Controlled on day 0	1 (50.0)	1 (50.0)	
Comorbidities			
Non-communicable diseases	6	3	
Acute diseases (e.g., diarrhea, pain)	2	5	
Lifestyle			
Tobacco use	8 (40.0)	12 (60.0)	0.43^a^
Alcohol use	11 (45.8)	13 (54.2)	0.80^a^
Practice of a healthy diet	15 (45.5)	18 (54.5)	0.63^a^
Practice of appropriate exercise	15 (46.9)	17 (53.1)	0.81^a^
Health education ever received			
Stop smoking	18 (46.2)	21 (53.8)	0.62^a^
Stop alcohol consumption	22 (50.0)	22 (50.0)	1.0^a^
Salt reduction	33 (54.1)	28 (45.9)	0.11^a^
Weight loss	16 (59.3)	11 (40.7)	0.32^a^
Regular exercise	31 (56.4)	24 (43.6)	0.062^a^
Number of medicines	2.2 ± 0.9	2.1 ± 1.0	0.49^c^
Herbal medicine			
Yes	5 (38.5)	8 (61.5)	0.36^a^
No	26 (54.2)	22 (45.8)	
Data unavailable	3 (42.9)	4 (57.1)	
Antihypertensive medicine (monotherapy)			
Enalapril	33	34	
Nifedipine	1	0	
Heart failure medicine (furosemide)	1	1	
Diabetes medicine	6	2	
Diazepam	2	6	
Antipyretic analgesic	12	8	
Vitamins	11	9	
Gastrointestinal drug	4	2	
Antibiotics	3	2	
Others	1	7	
Physical condition (day 0)			
Unwell	4 (50.0)	4 (50.0)	1.0^a^
Moderately unwell	21 (51.2)	20 (48.8)	
Moderately well	9 (50.0)	9 (50.0)	
Well	0 (0)	1 (100)	
Physical condition (day 7)			
Unwell	0 (0)	0 (0)	0.30^a^
Moderately unwell	0 (0)	3 (100)	
Moderately well	17 (54.8)	14 (45.2)	
Well	17 (50.0)	17 (50.0)	
Reason for consultation (day 0, multiple answers)			
Headache	22	19	
Dizziness	9	9	
Nausea	8	9	
Tiredness	8	5	
Loss of appetite	7	4	
Other acute symptoms	11	11	
Shortage of medicine	0	3	
Re-check	1	0	
Visit this hospital regularly (*n* = 42)	2	3	
Reason for not visiting hospital regularly (*n* = 37)			
Go to other health facility	15	9	
No need to continue taking medication	3	8	
Cost burden	0	1	
Come when unwell	1	0	
Where usually obtain hypertensive medicines (*n* = 42)			
Not regularly taking	2	5	
Private clinics	14	3	
District hospital	4	8	
District hospital and others	0	3	
Pharmacy	0	1	
Provincial hosp., private clinics	1	1	
When will be the next consultation (day 7)			
When condition worsens	26	16	0.03^a^
When medicine is complete	8	16	
Others	0	2	
Expenditure for treatment (LAK)	66,206 ± 68,955	98,824 ± 152,072	0.91^c^
Knows how to take medication	31 (50.8)	30 (49.2)	1.0
Do you think hypertension needs lifelong treatment?			
Yes	23 (63.9)	13 (36.1)	0.028^a^

^a^Fisher’s exact test

^b^Student’s *t*-test

^c^Mann–Whitney *U* test

All but one patient was prescribed enalapril for hypertension. Eight patients (11.8%) were prescribed diazepam, which might have been used for excessive hypertension [29]. The concomitant medications were those for heart failure (*n* = 2), anti-diabetics (*n* = 8), herbal medicine (*n* = 13), antipyretic analgesic (*n* = 20), vitamins (*n* = 20), gastrointestinal drugs (*n* = 6), antibiotics (*n* = 5), and others (*n* = 8; multiple answers allowed). Over half of the patients (*n* = 40, 58.8%) had no schedule for the next consultation; they stated that they would book the next consultation when they perceived their condition was worsening.

Members of the adherent group were significantly more likely to perceive that “hypertension needs lifelong treatment” (*p* = 0.028). However, there were no significant differences in BMI, blood pressure, lifestyle, number of medicines, and physical condition between the adherent and the non-adherent groups.

All patients received medication instructions (Table 4). Approximately half of the healthcare workers who provided instructions were medical doctors. Although the difference was not statistically significant, the adherent group was more likely to have received written instructions when medication was prescribed or dispensed, compared to the non-adherent group (79.4% vs. 55.8%, *p* = 0.068).

**Table 4:** Characteristics and practices of the healthcare workers who provided the medication and instructions regarding it for the patients

	Adherent group (*n* = 34)	Non-adherent group (*n* = 34)	*p*-value
Ethnic group			
Lao (Lao-Tai)	29 (51.8)	27 (48.2)	0.35^b^
Mangkong (Mon–Khmer)	0 (0)	3 (100)	
Phouthay (Lao-Tai)	5 (55.6)	4 (44.4)	
Profession			
Medical doctor	18 (50.0)	18 (50.0)	0.84^b^
Pharmacist	7 (50.0)	7 (50.0)	
Nurse	6 (66.7)	3 (33.3)	
Medical assistant	2 (40.0)	3 (60.0)	
Primary healthcare staff	1 (33.3)	2 (66.7)	
Laboratory assistant	0 (0)	1 (100)	
Experience (years), mean (SD)	2.7 (0.7)	2.7 (0.67)	0.95^a^
Medication instructions			
Provided oral instructions	34 (50.0)	34 (50.0)	–^b^
Provided written instructions	27 (58.7)	19 (41.3)	0.068^b^
Content of medication instructions			
Number of tablets per dose	34 (50.0)	34 (50.0)	–^b^
Number of doses per day	33 (50.0)	33 (50.0)	1.0^b^
Number of days of the regimen	21 (46.7)	24 (53.3)	0.61^b^
Effects of the medicine	20 (55.6)	16 (44.4)	0.47^b^
Side effects	19 (57.6)	14 (42.4)	0.33^b^
Importance of continuing to take medicines	20 (57.1)	15 (42.9)	0.33^b^
Confirmed understanding of the patient	17 (53.1)	15 (46.9)	0.81^b^

^a^Mann–Whitney *U* test

^b^Fisher’s exact test

^c^Student’s *t*-test

## Discussion

Half of the patients adhered closely to their prescribed antihypertensive medication regime. The level of medication adherence of this study (i.e., 50.0%) is similar to that reported in other studies conducted in a similar setting.

In a systematic review of 42 studies conducted among 19 developing countries, the mean adherence was 52.7%. The factors associated with poor adherence were low household income and socio-economic status, knowledge and beliefs regarding hypertension and its management, avoiding side effects of medications, cost of medication, use of herbal preparations, absence of symptoms, irregular follow-up, and dissatisfaction with the treatment and health services provided [30]. In the present study, there was likely no marked difference in socio-economic status between the adherent and the non-adherent groups, considering the nature of the occupations of the members of both groups. There was no significant difference in the use of herbal medicine, physical condition, health insurance, and treatment expenditure between groups. However, the cost tended to be greater in the non-adherent than in the adherent group. The physical condition on day 0 and day 7 and the reasons for consultation were similar between groups; more than 70% were moderately unwell or unwell and had acute symptoms on day 0, and more than 90% were moderately well or well on day 7. Most patients visited the hospital because of acute and/or severe symptoms. Of the patients, 33.8% were hospitalized after their consultation and one died within 7 days. Most patients (61.8%) stated that they would seek the next consultation when their condition deteriorated. Although most patients were diagnosed with grade 2 hypertension, they tended to seek consultation only when they felt unwell. Only two patients (2.9%) had controlled blood pressure (< 140 mmHg SBP and DBP < 90 mmHg; normal and high-normal hypertension), whereas 16.7% had controlled blood pressure in a national population-based survey conducted in Laos in 2013 [14]. This difference might be attributed to the population and study site difference between the prior and the present study.

The adherence reported in the present study is comparable with that reported in similar studies using the same measurement scale (i.e., MMAS-4). In a community-based cross-sectional study conducted in rural India [31], high adherence was seen in 46% of the patients. In primary healthcare network facilities in the Democratic Republic of Congo’s capital city, 54.2% of patients were non-adherent [24]. Among patients with hypertension in Pakistan, 29.3% exhibited poor adherence [23]. Among the inpatients of a secondary hospital in Uzbekistan, 36.8% exhibited adherence [28]. Of the outpatients with hypertension referred to rural healthcare centers in Iran, 24% exhibited adherence [22]. An adherence of 64.6% was found among outpatients with hypertension attending a university hospital in Ethiopia [27].

This study found that written instructions for the treatment of hypertension influenced medication adherence in poor, remote areas of a low- and middle-income country. In the present study, the adherent group tended to be more likely to have received written instructions when they were prescribed medications. The finding is consistent with a study conducted in the same region but for a different medication (i.e., adherence to malaria medication) [16]. It is necessary to promote the provision of written instructions in these areas; however, the following two issues should be considered and additional strategies developed and implemented accordingly. If there are differences in implementing written instruction among staff members, it may be necessary to eliminate these inconsistencies through retraining staff. Moreover, if there are differences in understanding written information by ethnicity and gender, it may be necessary to create and use simplified written information. In this study, 17 patients (25.0%) had not graduated from primary school (Table 1). Of the 17 patients, 6 patients (35.3%) were Mon–Khmer, an ethnic minority group, and 12 patients (70.6%) were women (data not shown). The low literacy of women, especially in poor and remote areas of Laos [32], is a point that requires consideration.

The district hospitals were the only public health facilities that provided medications for NCDs in the districts included in this study. Therefore, the minority groups living in remote areas may not be able to physically access the hospitals. Additionally, due to their lack of education, they may not understand the need to treat NCDs.

In addition, the adherent group was significantly more likely to perceive that “hypertension needs lifelong treatment”. Those who understood the importance of the medication appear to demonstrate greater adherence. Moreover, the adherent patients appeared to use private clinics more than public health facilities, whereas the lowest-priced generics in private clinics were 37.2% more expensive than those in public sector outlets [33]. Patients who attend private clinics have a wider choice of medicines, which might help them feel more comfortable with their treatment options. During the study period, only one or two antihypertensive medicines were available (enalapril and nifedipine were available in Xepone, enalapril 5 mg in Phin, enalapril 20 mg, and nifedipine 20 mg in Nong) of 10 essential antihypertensive medicines [34].

This study has the following limitations: (1) Since this was an exploratory study, the number of patients included in this study was relatively small. (2) The follow-up period may not have been long enough to adequately understand the level of medication adherence. In general, adherence to antihypertensive medication was measured among patients diagnosed with high blood pressure or who had been receiving treatment for over a month. However, since the adherence level of the patients who newly began antihypertensive medication and the other patients were the same, the former may have not fully understood the meaning of taking the medication. (3) Since data for this study were obtained from physicians who observed how other healthcare workers instructed the patients, it is likely that the staff provided patients with more careful instructions regarding medication. Due to these limitations, the level of medication adherence may have been overestimated.

## Conclusions

The medication adherence rate was 50% among patients with hypertension who visited a district hospital. Providing written instructions to patients would be effective for improving medication adherence.

## Data Availability

The datasets used and analyzed during the current study are available from the corresponding author on reasonable request.

## Abbreviations

Lao PDR: The Lao People’s Democratic Republic
NCDs: Non-communicable diseases
OPD: Outpatient department
ER: Emergency room
MMAS-4: Morisky Medication Adherence Scale (4-item)

## References

[1] World Health Organization. Noncommunicable diseases country profiles 2018. https://apps.who.int/iris/handle/10665/274512. Accessed 26 July 2021.

[2] Global Burden of Metabolic Risk Factors for Chronic Diseases Collaboration (2014). Cardiovascular disease, chronic kidney disease, and diabetes mortality burden of cardiometabolic risk factors from 1980 to 2010: a comparative risk assessment. Lancet Diabetes Endocrinol.

[3] Staessen JA, Gasowski J, Wang JG, Thijs L, Hond ED, Boissel J-P (2000). Risks of untreated and treated isolated systolic hypertension in the elderly: meta-analysis of outcome trials. The Lancet.

[4] Martiniuk AL, Lee CM, Lawes CM, Ueshima H, Suh I, Lam TH (2007). Hypertension: its prevalence and population-attributable fraction for mortality from cardiovascular disease in the Asia-Pacific region. J Hypertens.

[5] Stanaway JD, Afshin A, Gakidou E, Lim SS, Abate D, Abate KH (2018). Global, regional, and national comparative risk assessment of 84 behavioural, environmental and occupational, and metabolic risks or clusters of risks for 195 countries and territories, 1990–2017: a systematic analysis for the Global Burden of Disease Study 2017. The Lancet.

[6] Weber MA, Schiffrin EL, White WB, Mann S, Lindholm LH, Kenerson JG, et al. Clinical practice guidelines for the management of hypertension in the community. J Clin Hypertens. 2014;16(1):14–26. 10.1111/jch.12237. 10.1111/jch.12237 PMC8031779 24341872

[7] Corrao G, Parodi A, Nicotra F, Zambon A, Merlino L, Cesana G (2011). Better compliance to antihypertensive medications reduces cardiovascular risk. J Hypertens.

[8] Dragomir A, Côté R, Roy L, Blais L, Lalonde L, Bérard A (2010). Impact of adherence to antihypertensive agents on clinical outcomes and hospitalization costs. Med Care.

[9] Cutler RL, Fernandez-Llimos F, Frommer M, Benrimoj C, Garcia-Cardenas V (2018). Economic impact of medication non-adherence by disease groups: a systematic review. BMJ Open.

[10] Mazzaglia G, Ambrosioni E, Alacqua M, Filippi A, Sessa E, Immordino V (2009). Adherence to antihypertensive medications and cardiovascular morbidity among newly diagnosed hypertensive patients. Circulation.

[11] Bowry AD, Shrank WH, Lee JL, Stedman M, Choudhry NK (2011). A systematic review of adherence to cardiovascular medications in resource-limited settings. J Gen Intern Med.

[12] Lao People’s Democratic Republic. Report on STEPS survey on non communicable diseases risk factors in Vientiane Capital city, Lao PDR. 2010. https://www.who.int/ncds/surveillance/steps/laos/en/. Accessed 26 July 2021.

[13] Lao People's Democratic Republic, Ministry of Health. National Multisectoral Action Plan for the Prevention and Control of Noncommunicable Diseases 2014–2020 (LAOSMAP-NCD). 2014. https://www.iccp-portal.org/system/files/plans/LAO_NCD_LAO_B3_natl_multisec_NCD_plan_2014-2020.pdf. Accessed 26 July 2021.

[14] Pengpid S, Vonglokham M, Kounnavong S, Sychareun V, Peltzer K (2019). The prevalence, awareness, treatment, and control of hypertension among adults: the first cross-sectional national population-based survey in Laos. Vasc Health Risk Manag.

[15] Burnier M, Egan BM (2019). Adherence in hypertension. Circ Res.

[16] Takahashi E, Nonaka D, Iwagami M, Phoutnalong V, Chanthakoumane K, Kobayashi J (2018). Patients' adherence to artemisinin-based combination therapy and healthcare workers' perception and practice in Savannakhet province, Lao PDR. Trop Med Health.

[17] World Health Organization. The Lao People’s Democratic Republic health system review. Health Systems in Transition. 2014;4(1). https://apps.who.int/iris/bitstream/handle/10665/207762/9789290616481_eng.pdf;sequence=1. Accessed 26 July 2021.

[18] JICA Data Collection Survey on Health Sector. Country report: Lao People's Democratic Republic. 2012. https://openjicareport.jica.go.jp/pdf/12085205.pdf. Accessed 26 July 2021.

[19] Unger T, Borghi C, Charchar F, Khan NA, Poulter NR, Prabhakaran D (2020). 2020 International Society of Hypertension Global Hypertension Practice Guidelines. Hypertension.

[20] Morisky DE, Green LW, Levine DM (1986). Concurrent and predictive validity of a self-reported measure of medication adherence. Med Care.

[21] Lam WY, Fresco P (2015). Medication adherence measures: an overview. Biomed Res Int.

[22] Kamran A, Sadeghieh Ahari S, Biria M, Malepour A, Heydari H (2014). Determinants of patient's adherence to hypertension medications: application of health belief model among rural patients. Ann Med Health Sci Res.

[23] Arshad AR (2015). Frequency of poor adherence to antihypertensive treatment and an analysis of clinico-demographic correlates. J Coll Physicians Surg-Pak JCPSP.

[24] Lulebo AM, Mutombo PB, Mapatano MA, Mafuta EM, Kayembe PK, Ntumba LT (2015). Predictors of non-adherence to antihypertensive medication in Kinshasa, Democratic Republic of Congo: a cross-sectional study. BMC Res Notes.

[25] Venkatachalam J, Abrahm SB, Sinhypertgh Z, Stalin P, Sathya GR (2015). Determinants of patient's adherence to hypertension medications in a rural population of Kancheepuram District in Tamil Nadu, South India. Indian J Community Med.

[26] Suleiman A (2010). Elevated blood pressure among patients with hypertension in General Hospital of Penang, Malaysia: does poor adherence matter?. Int J Pharm Pharm Sci.

[27] Ambaw AD, Alemie GA, Yohannes SMW, Mengesha ZB (2012). Adherence to antihypertensive treatment and associated factors among patients on follow up at University of Gondar Hospital, Northwest Ethiopia. BMC Public Health.

[28] Malik A, Yoshida Y, Erkin T, Salim D, Hamajima N (2014). Hypertension-related knowledge, practice and drug adherence among inpatients of a hospital in Samarkand, Uzbekistan. Nagoya J Med Sci.

[29] Grossman E, Nadler M, Sharabi Y, Thaler M, Shachar A, Shamiss A (2005). Antianxiety treatment in patients with excessive hypertension. Am J Hypertens.

[30] Dhar L, Dantas J, Ali M (2017). A systematic review of factors influencing medication adherence to hypertension treatment in developing countries. Open J Epidemiol.

[31] Balasubramanian A, Nair SS, Rakesh PS, Leelamoni K (2018). Adherence to treatment among hypertensives of rural Kerala, India. J Fam Med Prim Care.

[32] Lao Statistics Bureau. Results of Population and Housing Census 2015 (English Version). 2016. https://lao.unfpa.org/en/publications/results-population-and-housing-census-2015-english-version. Accessed 31 Aug 2021.

[33] Lao People’s Democratic Republic. Medicine prices, availability, affordability and price components in the Lao People’s Democratic Republic, 2013 (English version). http://www.fdd.gov.la/download/contents_documents/1411701061Laos%20summary%20report%20drug%20price_capability_component_English%20version.pdf. Accessed 26 July 2021.

[34] Lao People’s Democratic Republic. List of essential medicines of Lao PDR. 2019. http://www.fdd.gov.la/download/contents_documents/Final_of%20EML9th_Latest_Updated-01-August-2019.pdf. Accessed 26 July 2021.

